# Effect of various interventions for smoked tobacco cessation among Indians in Chhattisgarh

**DOI:** 10.6026/973206300210225

**Published:** 2025-02-28

**Authors:** Milind Wasnik, Bhavna Dave, Virendra Vadher

**Affiliations:** 1 Department of Public Health Dentistry, KM Shah Dental College and Hospital, Vadodara, Sumandeep Vidyapeeth Deemed to be University, Piparia, Vadodara, Gujarat, India; 2 Department of Paediatric and Preventive Dentistry, KM Shah Dental College, Vadodara, Gujarat state, India; 3 Government Dental College, Raipur, Chhattisgarh, India

**Keywords:** Tobacco cessation, nicotine replacement therapy, behavioral therapy, randomized controlled trial, mobile health

## Abstract

A prospective, single-blind, randomized controlled trial was conducted among 150 adult tobacco users attending the Tobacco Cessation Centre, Government Dental College, Raipur and Chhattisgarh, India. Participants were randomized into three groups: Group I (NRT alone), Group II (NRT + counseling) and Group III (NRT + mCessation). Interventions lasted 12 weeks, with follow-ups at 1 and 3 months. The overall quit rate was 34%. Group II demonstrated the highest success rate (44%), followed by Group III (30%) and Group I (28%). Significant reductions in nicotine dependence, CO levels and cigarette consumption were observed in all groups, with Group II showing the most marked improvements. Behavioral counseling combined with pharmacotherapy is the most effective strategy for smoking cessation.

## Background:

The tobacco situation in the world is unique because of the vast spectrum of tobacco products available for smoking as well as smokeless use [1]. The global tobacco epidemic is characterized by a vast array of tobacco products used for smoking and smokeless purposes, affecting nearly 1.3 billion people worldwide, 80% of who live in low- and middle-income countries. Tobacco use claims over 8 million lives annually [2]. In India, the Global Adult Tobacco Survey (GATS-2) [3] reveals that 28.6% of adults use tobacco, with 87% being daily users. The prevalence is higher among men (42.4%) than women (14.2%) and more pronounced in rural areas (32.5%) compared to urban areas (21.2%). Among daily users aged 20-34, 35.8% started before 18, underscoring early initiation as a key factor in prolonged use and addiction. Smoking products include bidis, cigarettes, cigars, hukkah and electronic cigarettes. GATS-2 data indicates that 10.7% of Indian adults smoke, with 80% being daily smokers. Smoking prevalence is significantly higher among men (19%) compared to women (2%) and is more common in rural areas (11.9%) than urban areas (8.3%). Smokeless tobacco is even more prevalent, with 36% of adults using like khaini and tobacco for oral application. In Chhattisgarh, 53.7% of men and 24.6% of women either smoke or use smokeless tobacco. Notably, tobacco use among 15-17-year-olds decreased from 15.9% in GATS-1 to 9.3% in GATS-2, with the mean initiation age rising from 16.2 to 18.5 years [4]. A key component of quitting smoking is behavioural therapy. Cognitive behavioural therapy (CBT) and motivational interviewing (MI) are two types of individual counselling that have been shown to be successful in treating addiction triggers and improving motivation [5, 6]. Peer support is offered by group treatment and accessibility is guaranteed by telephone counselling via quit lines [7]. Digital tools have become more popular, such as mobile health (mHealth) interventions like text messaging apps and smartphone apps. These programs use evidence-based strategies to increase quit rates by providing tracking features, motivational content and individualized assistance. Apps' social support features increase their efficacy even further [8, 9-10]. Behavior-based approaches are supplemented with pharmaceutical interventions. Nicotine Replacement Therapy (NRT) provides nicotine without the use of hazardous tobacco compounds, thereby decreasing withdrawal symptoms. Gum and patches are among the types that boost stopping success rates by 50-60% [11]. It works considerably better when NRT forms are combined. Antidepressant bupropion lessens post-cessation weight gain and withdrawal symptoms [12]. By blocking the rewarding effects of nicotine and reducing withdrawal symptoms, Varenicline, a partial agonist at nicotinic receptors, doubles the success rate of quitting compared to a placebo. Research supports the advantages of varenicline over single-form NRT and bupropion [13]. The combination of pharmaceutical and behavioural therapies leads to increased success rates. For example, thorough assistance is offered when counselling is combined with drugs such as varenicline [13]. The Clinical Practice Guideline promotes this combination strategy, emphasizing how well it works to assist people in quitting. In order to battle the global tobacco epidemic and lessen the significant burden of avoidable diseases and untimely deaths, these multifaceted tactics are essential [14-15]. Therefore, it is of interest to evaluate the effect of various interventions for smoked tobacco cessation among Indians in Chhattisgarh.

## Methods and Materials:

## Study design:

Participants in this prospective, single-blind, randomised controlled interventional study were those who visited the Government Dental College's Tobacco Cessation Centre in Raipur, Chhattisgarh. The effectiveness of three tobacco cessation therapies was evaluated in the study: nicotine replacement therapy (NRT), counseling and NRT and mCessation and NRT. Follow-ups were held at one and three months after the participants got their individual interventions throughout a three-month period. The Declaration of Helsinki's ethical guidelines were adhered to when conducting this study. Ethical approval was obtained from the Institutional Ethics Committee of Government Dental College, Raipur, with reference number IEC: ECR/6912/GDC/CG/2019 and from Sumandeep Vidyapeeth, with reference number SVIFC/ON/DenHPhD/Nov/22/13. Written informed consent was obtained from all participants prior to their inclusion in the study.

## Sample size and randomization:

A sample size of 105 participants was determined using G*Power 3.9.1.2, considering a 5% alpha error, 20% beta error and 10% clinical difference. To account for dropouts, 50 participants were included in each group. 150 Participants were randomized equally into the three intervention groups using block randomization with variable block sizes of three.

## Eligibility criteria:

## Inclusion criteria:

[1] Outpatients at the hospital, within the age band of 18-60 years.

[2] Participants who remained current users of "smoking form" of tobacco.

[3] Participants who confirmed to remain at their current address till the completion of the study.

[4] Participants who able to provide written informed consent and understand the study protocol.

[5] Participants who can access to the mobile phone.

## Exclusion criteria:

[1] Terminal ailment (prognosis <12 months)

[2] Pregnant women, lactating mothers are excluded.

[3] Patients under psychiatric care

[4] History of hypersensitive reactions toward nicotine or menthol

[5] Previous admission into the similar study

[6] Patients using both smoking and smokeless variants of tobacco.

Participants underwent baseline assessments, including demographic data collection, tobacco use history and nicotine dependency levels using the Fagerström Test for Nicotine Dependence (FTND). Clinical evaluations included exhaled carbon monoxide (CO) measurements and urine cotinine levels to establish baseline tobacco exposure. The intervention period lasted 12 weeks and participants were allocated to one of three groups: Group I-NRT Group: Nicotine gum was prescribed based on smoking intensity: 4 mg/day for ≥25 cigarettes/day and 2 mg/day for <25 cigarettes/day. Gum usage was scheduled as follows: Weeks 1-6: 1 piece every 1-2 hours; Weeks 7-9: 1 piece every 2-4 hours; Weeks 10-12: 1 piece every 4-8 hours. A maximum of 24 pieces per day was permitted. Group II-NRT + Counseling Group: Participants received NRT as described above, along with 30 minutes of personalized tobacco cessation counseling using the 5 A's (Ask, Advise, Assess, Assist, Arrange) and 5 R's (Relevance, Risks, Rewards, Roadblocks, Relapse) frameworks. Group III- NRT + mCessation Group: In addition to NRT, participants were enrolled in the mCessation program, a mobile-based initiative providing motivational and cessation support through text messages. Data collection included a structured questionnaire that gathered demographic details, tobacco use history and smoking habits, such as frequency and duration of use. Nicotine dependency levels were evaluated using the Fagerström Test for Nicotine Dependence (FTND), categorizing participants into low, moderate, or high dependency groups. Exhaled CO levels were measured with a handheld monitor to validate self-reported smoking behavior, while urine cotinine testing provided an objective measure of nicotine intake. Follow-ups were carried out at 1and 3months to assess various elements of the interventions. Urine cotinine analysis and exhaled carbon monoxide (CO) monitoring were used to evaluate side effects, adherence to the interventions and decreases in tobacco use at the 1-month follow-up. The goal of the 3-month follow-up, which concluded the intervention phase, was to assess the interventions' efficacy using self-reported data as well as biochemical verification. Biochemical measurements were used to evaluate durable behaviour improvements and relapse rates.

## Results:

In high-prevalence areas like Chhattisgarh, quitting smoking is still a major public health concern. In this study, 150 individuals were randomly assigned to three groups and the effectiveness of three intervention modalities for quitting smoking tobacco was evaluated. Important information about their relative efficacy was revealed by follow-ups conducted at one and three months. Information about the research participants' demographics, such as their mean age, marital status and gender distribution, is included in Table 1. Table 2 shows information on the participants' smoking behaviors, including how many cigarettes they smoked each day and how long they smoked, is included.

Individuals were divided into "Quit" and "Still Using" groups and the treatments were evaluated according to how well they assisted individuals in quitting smoking. Group II had the greatest success rate (44%), followed by Group III (30%) and Group I (28%). In all, 34% of individuals were able to stop smoking. Regarding sustained tobacco usage, 66% of participants in all categories continued to smoke, underscoring the on-going problem of tobacco addiction.

## Group-specific outcomes:

Group I had a quit rate of 28%, while 72% of individuals continued to smoke. This group had the lowest quit rate of the three, indicating that the intervention utilized may need to be modified or improved to have better results. In contrast, Group II demonstrated the highest quit rate at 44%, with 56% of participants continuing to smoke. This suggests that the intervention in Group II was the most effective strategy and holds promise for wider application. Group III had a quit rate of 30%, with 70% of participants continuing to smoke. The results for this group were moderate, showing a slightly better quit rate than Group I but significantly lower than Group II.

## Interpretation of findings:

At the 1-month follow-up, adherence to the interventions, side effects and reductions in tobacco use were assessed through urine cotinine analysis and exhaled carbon monoxide (CO) monitoring (Table 4). The 3-month follow-up, marking the end of the intervention phase, focused on evaluating the effectiveness of the interventions using both biochemical verification and self-reported data (Table 3, Table 4 and Table 5). Table 3 provides a comparison of nicotine dependence scores at baseline and at 3 months across the three intervention groups, highlighting significant changes. Table 4 compares the carbon monoxide levels measured at baseline and at 3 months, reflecting reductions in smoking intensity. Table 5 summarizes the change in the number of cigarettes smoked over the intervention period. Exhaled CO levels were measured with a handheld monitor to validate self-reported smoking behavior (Figure 1 - (see PDF) and Table 4), while urine cotinine testing provided an objective measure of nicotine intake (Table 5).

## Discussion:

Behavioral counseling has proven effective in facilitating smoking cessation through addressing psychological, substance, social and behavioral aspects of nicotine dependence. Effective interventions to reduce iron deficiency systematic reviews [16] have highlighted the effectiveness of different types of counseling (*e.g.*, individual, group and telephone-based). For example, one study found that individual contact with cessation specialists resulted in a mean quit rate of 11.4% v 7.7% for minimal contact interventions. Counseling methods that are more intensive and involve more frequent sessions, along with individualized support, are associated with higher quit rates. This has also been supported by García-Gómez *et al.* (2019) [17] in that the intensity of counseling impacts cessation rates. Behavioral counseling emphasizes motivation and provides coping mechanisms to deal with withdrawal symptoms and triggers and is, therefore, a key part of tobacco cessation programs. Medications, including nicotine replacement therapy (NRT), varenicline and bupropion, have been shown to substantially increase quitting rates compared to placebo treatments. As an example, a Cochrane review [18], reported that varenicline (a partial nicotine receptor agonist) produced an odds ratio (OR) of sustained abstinence when used alone of 2.83 and an odds ratio of up to 5.75 when used with NRT. Available in a variety of forms, including patches, chewing gum and lozenges, NRT is widely known for its role in reducing withdrawal symptoms and facilitating gradual abstinence from nicotine. However, in the current study, Group I, who relied solely on NRT, had the lowest abstinence rate, supporting the idea that pharmacotherapy alone is not sufficient to sustain smoking cessation. This result is consistent with studies that highlight that pharmacotherapy is most effective when combined with behavioral interventions [6]. Safety also plays an important role in pharmacotherapy. Most options, including NRT and varenicline, have minimal side effects, whereas bupropion is associated with a higher risk of severe side effects compared to placebo [19]. Patient selection and close monitoring are therefore essential to ensure the safety and effectiveness of these pharmacological interventions.

The combination of behavioral counseling and pharmacotherapy appears to be the most effective strategy for tobacco cessation, as evidenced by the superior quit rates in Group II of this study. Participants who received both NRT and behavioural counselling achieved significantly higher rates of abstinence compared to those who received either intervention alone. These findings are consistent with previous studies [20, 21] found that combining behavioural support with varenicline led to markedly improved cessation outcomes. The synergistic effect of combining these approaches addresses both the physiological dependency on nicotine and the psychological behaviours associated with smoking. This integrated strategy not only enhances quit rates but also helps sustain abstinence over time [22]. It reinforces the importance of implementing comprehensive cessation programs that cater to the multifaceted nature of tobacco addiction. In recent years, mobile health (mHealth) interventions have emerged as an innovative approach to smoking cessation. Mobile apps and text message-based programs offer convenience, scalability and personalization. However, the results for Group III, which included NRT with mobile cessation support, were moderate in this study, highlighting potential limitations in participant engagement or intervention design. Although studies [10, 23] have reported the efficacy of mHealth interventions, challenges such as user adherence and tailoring interventions to diverse populations must be addressed to maximize their potential. Recent advancements in mobile health (mHealth) interventions have shown promise in supporting smoking cessation. A study [24] published in 2023 reviewed 39 randomized controlled trials and found that eHealth interventions, particularly mHealth approaches, might promote smoking cessation, although their effectiveness may diminish over time. Additionally, researchers [25] at the University of Bristol developed a smart watch app that detects smoking-related hand movements and provides real-time supportive messages, receiving positive feedback from participants. However, the effectiveness of smartphone app-based interventions remains equivocal. A systematic review and meta-analysis [24] indicated that while these interventions are widely used, the evidence for their effectiveness is currently controversial, suggesting the need for more personalized and well-designed interventions. The findings of this study underscore the need for a multimodal approach to tobacco cessation. Integrating behavioral counseling with pharmacotherapy not only enhances cessation rates but also addresses the multifaceted nature of nicotine dependence. The moderate results for mobile smoking cessation highlight the need for further innovation and optimization in digital health interventions. Future programs should focus on personalized and culturally appropriate strategies to maximize engagement and effectiveness.

## Limitations and future directions:

This study is limited by a relatively small sample size and a short follow-up period. Future studies should include larger and more diverse populations and extend follow-up periods to assess long-term abstinence rates. Additionally, investigating the specific elements of behavioral counseling that contributed to Group II's success may help refine the intervention for broader implementation.

## Conclusion:

The importance of combining behavioral counseling and pharmacotherapy for sustained smoking cessation is highlighted. Pharmacotherapy and mobile smoking cessation support showed moderate effectiveness, whereas the integrated approach showed better results. These findings contribute to a growing evidence base supporting comprehensive and diverse smoking cessation strategies tailored to the individual needs of smokers in different populations.

## Ethical statement:

This study was conducted following the ethical principles outlined in the Declaration of Helsinki. Ethical approval was obtained from the Institutional Ethics Committee of Government Dental College, Raipur, with reference number IEC: ECR/6912/GDC/CG/2019 and from Sumandeep Vidyapeeth, with reference number SVIFC/ON/DenHPhD/Nov/22/13.

## Funding:

This study was funded by the National Tobacco Control Programme (NTCP) and the Department of Health and Family Welfare, Government of Chhattisgarh. The funding supported the study's execution but did not influence its design, data collection, analysis, or manuscript preparation. The authors sincerely thank these organizations for their valuable support.

## Author contribution:

All authors contributed significantly to the study. Milind Wasnik conceptualized the study design, supervised data collection and performed the primary analysis. Bhawna Dave was responsible for literature review, data interpretation and drafting the manuscript. Virendra Vadher provided critical revisions and coordinated the submission process. All authors reviewed and approved the final manuscript.

## Study registration:

This study was registered in the Clinical Trials Registry - India (CTRI) with the acknowledgment number REF/2023/03/064628. The registration ensures adherence to ethical standards and transparency in the study's design and implementation.

## Figures and Tables

**Table 1:** Details of the students

**Characteristics**		**Frequency**
Gender	Male	150 (100.00)
	Female	0
Mean Age	Group I	36.16 ±13.34
	Group II	37.18 ±13.57
Marital Status	Group III	34.98 ±13.50
	Married	91 (60.6)
	Unmarried	59 (39.4)

**Table 2:** Amount of cigarette smoking and duration of study participants

**Characteristics**		**Frequency**
Smoking amount per Day	< 5	95 (63.33)
(Cigarettes / Day)	10-May	34 (22.66)
	15-Oct	14 (9.33)
	> 15	7 (4.66)
Year of Smoking	< 5	57 (38)
	10-May	39 (26)
	> 10	54 (36)

**Table 3:** Comparison of groups for change in nicotine dependence score

**Groups**		**At Baseline**	**3 Months**	**Difference change**	**P**
Group I	NRT	3.30±2.17	1.97±1.74	1.32±0.82	0.001
Group II	NRT with Counselling	2.67±1.60	0.53±0.82	2.14±1.17	0.001
Group III	NRT with m-cessation	2.63±1.63	1.14±1.29	1.49±1.00	0.001
Group I Vs II < 0.05					
Group I Vs III < 0.05					
Group II Vs III > 0.05					

**Table 4:** Comparison of groups for change in amount of Carbon Monoxide level

**Groups**		**At Baseline**	**3 Months**	**Difference change**	**P**
Group I	NRT	6.31±5.55	3.64±3.94	2.67±1.94	0.001
Group II	NRT with Counselling	6.02±3.87	1.24±1.75	4.78±2.70	0.001
Group III	NRT with m-cessation	6.45±5.21	3.50±3.99	2.95±1.55	0.001
Group I Vs II < 0.05					
Group II Vs III < 0.05					
Group I Vs III > 0.05					

**Table 5:** Comparison of groups for change in amount of Cigarettes smoked

**Groups**		**At Baseline**	**3 Months**	**Difference change**	**P**
Group I	NRT	5.82±4.75	3.04±3.57	2.78±1.60	0.001
Group II	NRT with Counselling	5.24±3.81	1.08±1.48	4.16±2.78	0.001
Group III	NRT with m-cessation	5.85±4.82	2.77±3.69	3.08±1.51	0.001
Group I Vs II < 0.05					
Group II Vs III < 0.05					
Group I Vs III > 0.05					
